# Regional nodal irradiation for breast cancer using volumetric modulated arc therapy: Echocardiographic functional outcomes

**DOI:** 10.21203/rs.3.rs-2908730/v1

**Published:** 2023-06-08

**Authors:** Anthony Yu, Charlie White, Zhigang Zhang, Jennifer Liu, Erin Gillespie, Beryl McCormick, Atif Khan, Richard Steingart, Simon Powell, Oren Cahlon, Lior Braunstein

**Affiliations:** Memorial Sloan Kettering Cancer Center; Memorial Sloan Kettering Cancer Center; Memorial Sloan Kettering Cancer Center; Memorial Sloan Kettering Cancer Center; Memorial Sloan Kettering Cancer Center; Memorial Sloan Kettering Cancer Center; Memorial Sloan Kettering Cancer Center; Memorial Sloan Kettering Cancer Center; Memorial Sloan Kettering Cancer Center; Memorial Sloan Kettering Cancer Center; Memorial Sloan Kettering Cancer Center

**Keywords:** breast cancer, regional nodal irradiation, volumetric modulated arc therapy, radiotherapy, echocardiogram

## Abstract

**Introduction:**

Regional nodal irradiation (RNI) for breast cancer yields improvements in disease outcomes, yet comprehensive target coverage often increases cardiac radiation (RT) dose. Volumetric modulated arc therapy (VMAT) may mitigate high-dose cardiac exposure, although often increases the volume of low-dose exposure. The cardiac implications of this dosimetric configuration (in contrast to historic 3D conformal techniques) remains uncertain.

**Methods:**

Eligible patients receiving adjuvant RNI using VMAT for locoregional breast cancer were prospectively enrolled on an IRB-approved study. Echocardiograms were performed prior to RT, at the conclusion of RT, and 6-months following RT. Echocardiographic parameters were measured by a single reader (AY) and measures were compared pre- and post-RT via the Wilcoxon rank sum test. Changes in echocardiographic parameters over time were compared to mean and max heart doses via the Spearman correlation test.

**Results:**

Among 19 evaluable patients (median age 38), 89% (n=17) received doxorubicin and 37% (n=7) received trastuzumab/pertuzumab combination therapy. All patients received VMAT-based whole-breast/chest-wall and regional nodal irradiation. Average mean heart dose was 456cGy (range 187–697cGy) and average max heart dose was 3001cGy (1560–4793cGy). Among salient echocardiographic parameters, no significant decrement in cardiac function was observed when comparing pre-RT to 6-months post-RT: mean left ventricular ejection fraction (LVEF) was 61.8 (SD 4.4) pre-RT and 62.7 (SD 3.8) 6-months post-RT (p=0.493); mean global longitudinal strain (GLS) was −19.3 (SD 2.2) pre-RT and −19.6 (SD 1.8) 6-months post-RT (p=0.627). No individual patient exhibited reduced LVEF or sustained decrement in GLS. No correlations were observed for changes in LVEF or GLS when compared to mean or maximum heart doses (p>0.1 for all).

**Conclusions:**

VMAT for left-sided RNI yielded no significant early decrement in echocardiographic parameters of cardiac function, including LVEF and GLS. No patient exhibited significant LVEF changes, and none exhibited sustained decrements in GLS. VMAT may be a reasonable approach to cardiac avoidance in patients requiring RNI, including those receiving anthracyclines and HER2-directed therapy. Larger cohorts with longer follow-up will be needed to validate these findings.

## Introduction

Over 3 million cancer survivors in the United States have undergone treatment comprising some measure of cardiac radiotherapy (RT) exposure. Several series have demonstrated that chest/breast radiotherapy may result in increased cardiovascular (CV) morbidity, including cardiomyopathy, coronary disease, and valvular dysfunction^1–8^. Moreover, whereas the traditional understanding of RT-associated cardiotoxicity assumed a long latency period for the manifestation of cardiac events via accelerated fibrotic changes, recent studies suggest that early cardiotoxicity can be observed^1,3^.

Many epidemiologic analyses that associate RT with CV toxicity are based on outdated RT delivery techniques (e.g. conventional, 2D, or 3D conformal RT) and systemic therapies. The applicability of these findings to current RT approaches and systemic agents remains unknown, and uncertainty persists regarding the mechanisms and predictors of cardiotoxicity^9^. Moreover, with the implementation of advanced RT delivery technologies (e.g. surface imaging, respiratory gating, protons, etc.), improvements in cardiac dose reduction remain of hypothetical benefit with limited clinical indications of long-term superiority^10^. Further elucidation of RT-associated CV toxicity is of particular importance given a series of landmark studies that prompted increased utilization of regional nodal irradiation (RNI)^11,12^. This comprehensive breast RT approach typically yields elevated cardiac exposure due to proximity of the internal mammary nodal (IMN) target to the heart.

Volumetric modulated arc therapy (VMAT) is one of several approaches designed to reduce cardiac exposure from RNI (and thereby mitigate putative CV risk)^13–15^, yet the cardiac implications of this technique are unknown. Indeed, while VMAT improves the conformality of high-dose RT regions, it necessarily exposes more non-target tissue to low-doses of radiation. This typically reduces the maximum heart dose as intended, while simultaneously increasing the mean heart dose (MHD) with, as yet, indeterminate consequences.

Echocardiography is a primary modality for assessing structural and functional changes secondary to the cardiotoxic effects of RT. Myocardial strain imaging using speckle-tracking echocardiography is an established tool for quantitative measurement of cardiac contractile function.^16^ Myocardial strain parameters such as global longitudinal strain (GLS) are more sensitive compared with left ventricular ejection fraction (LVEF) for detecting early stages of cardiotoxicity related to radiotherapy and have established prognostic value in multiple treatment settings.^17–19^ Myocardial work indices are novel echocardiography-based measures that have been proposed to provide complementary assessment of cardiac function over GLS, however the value of MW indices to detect the cardiotoxic effects of radiotherapy is unknown.

Thus, we undertook a prospective single-arm cohort study to quantify the longitudinal change in myocardial strain and myocardial work indices among breast cancer patients receiving RNI via VMAT. Imaging was conducted pre-RT, at the end of RT, and 6-months following RT, to assess for putative subclinical changes in cardiac function arising from treatment.

## Methods

### Study Design

This was a single-arm prospective cohort study designed to evaluate changes in myocardial function based on 2D echocardiographic indices among breast cancer patients receiving left-sided RNI using VMAT. Patients with invasive locoregional breast cancer (i.e. non-metastatic) were prospectively screened and approached for participation on this IRB-approved study by the treating physician(s). Eligible and consenting patients were enrolled at the time of treatment consent for adjuvant RNI using VMAT (inclusive of RT to the axilla, supraclavicular fossa, internal mammary nodal basin and chestwall/breast to a total dose of 50Gy in 25 fractions). Thereafter, echocardiograms were performed at three timepoints: prior to RT (typically between the time of simulation and treatment start), at the conclusion of RT (typically within 1–2 weeks of the last RT fraction), and 6-months following RT. All study patients provided fully-informed written consent prior to participation. This study protocol was approved by the Memorial Sloan Kettering Cancer Center institutional review board and was compliant with the Health Insurance Portability and Accountability Act.

### Echocardiography

2D and Doppler echocardiography was performed using a Vivid E9 ultrasound scanner (GE Medical Systems, Horten, Norway) at the following timepoints: pre-RT, post-RT, and 6 months post-RT. LVEF was calculated from the apical 4- and 2-chamber views using a modified Simpson biplane method according to the American Society of Echocardiography guidelines.^20^ During the standard 2D echocardiogram, apical 2-, 3-, and 4- chamber views and short-axis views at the midpapillary level were acquired at a frame rate of 40–80 frames per second. Speckle tracking strain analysis was performed offline to calculate peak systolic global longitudinal (GLS), circumferential (GCS) and radial (GRS) strain (Echopac, GE Medical, Milwaukee, WI) as previously described.^21^ All strain analysis was performed by a board-certified cardiologist (A.Y.), independent of the clinical interpretation of the 2D echocardiogram. Strain analysis was conducted in a manner blinded to patient radiotherapy plan or dosimetry.

Myocardial work indices were measured with commercially available software (GE Echopac), as previously described.^22^ The peak systolic LV pressure was estimated based upon brachial systolic blood pressure measurement. Timing of mitral and aortic valve opening and closure, measured by pulse-wave Doppler and confirmed by 2D assessment in the apical long axis view, were used to define isovolumic and systolic ejection periods. The software then used the peak systolic LV pressure, valvular timing events, and GLS measurements to automatically generate left ventricular pressure strain loops. The following indices were then calculated: 1) global work index (GWI), which represents the total area of the pressure strain loop from mitral valve closure to opening; 2) global constructive work (GCW), which represents myocardial work during systolic shortening and negative work (i.e. work performed by the blood on the ventricle) during lengthening in isovolumic relaxation; 3) global wasted work (GWW), which represents negative work during lengthening in systole and myocardial work during shortening in isovolumic relaxation; and 4) global work efficiency (GWE), which is calculated as GCW divided by the sum of GCW and GWW.

Inter-observer and intra-observer variability for MW indices was assessed in 20 randomly selected studies by the intraclass correlation coefficient (ICC) and standard error of measurement. For inter-observer variability, studies were remeasured by two observers blinded to one another’s findings (A.Y. and J.L.). For intra-observer variability, studies were remeasured by the same observer at different points in time.

### Statistical Analysis

Continuous measures were summarized as mean and standard deviation, and categorical measures were summarized as frequency and percent. Differences in echocardiographic parameters between timepoints (pre-RT, post-RT, and 6 months post-RT) were explored by analysis of variance for repeated measures. Differences between the pre-RT and post-RT timepoints were further compared using the Wilcoxon rank sum test. The Spearman correlation test was used to assess the effect of mean and max heart doses on changes in echocardiography-based parameters from pre-RT to 6 months post-RT.

## Results

### Patient and treatment characteristics

From 2018 to 2020, 19 patients were enrolled on this prospective study (median age 38, range 26 – 61) (Table 1). Of these, 16 (84%) underwent mastectomy, 3 (16%) underwent breast conservation, 4 (21%) had sentinel lymph node biopsy alone, and 15 (79%) had a complete axillary lymph node dissection. Axillary evaluations yielded a median of 2 positive nodes (range 0 −18), after recovering a median of 16 nodes in total (range 2–27). Most patients received doxorubicin-based chemotherapy (n = 17; 89%), and 7 (39%) further received HER2-directed therapy comprising trastuzumab and pertuzumab. All patients received RNI using the VMAT technique, yielding a median mean heart dose of 475cGy (range 187 – 698) and a median maximum heart dose of 2999cGy (range 1560–4793). No patients exhibited cardiac events over the follow-up period and none were admitted for cardiac diagnoses.

### Longitudinal changes in cardiac structure and function

Echocardiographic parameters of cardiac structure and function over time are shown in Table 2. LVEF was normal at pre-RT (61.8% ± 4.4%) and did not significantly change post-RT (62.2% ± 4.3%) or at 6-months post-RT (62.7 % ± 3.8%). Similarly, no changes in diastolic indices were observed during or after RT. We then measured indices of myocardial strain to assess for subclinical changes in cardiac function during radiotherapy. There were no significant differences from pre-RT to 6 months post-RT in GLS (−19.3% ± 2.2% vs. −19.6% ± 1.8%, P = 0.627), GCS (−16.6% ± 3.6% vs. −16.2% ± 3.5%, P = 0.986), or GRS (45.8 ± 23.9% vs. 43.3 ± 15.7%, P = 0.958).

Myocardial work indices of the study population over time are shown in Table 3. All patients had normal myocardial work indices pre-RT based upon published normal reference values.^23^ There were no significant differences in GWI, GCW, GWW, or GWE from pre-RT to 6-months post-RT.

### Echocardiographic parameters and radiotherapy dose

We evaluated for associations between cardiac dosimetry and longitudinal changes in echocardiographic parameters of cardiac function during the study period (from pre-RT to 6-months post-RT). There was no correlation between change in LVEF (pre-RT to 6 months post-RT) and mean (r = −0.02, P = 0.931) or max (r = −0.02, P = 0.948) heart dose. Similarly, we found no correlation between change in GLS (pre-RT to 6-months post-RT) and mean (r=0.35, P=0.15) or max (r = 0.18, P=0.475) heart dose.

We then evaluated for associations between cardiac dosimetry and longitudinal changes in myocardial work indices. There was a significant correlation between mean heart dose and decline in GWI from pre-RT to 6 months post-RT (r= −0.51, P = 0.037), but not max heart dose and GWI (r=−0.13, P=0.619). GWI declined from pre-RT to 6 months post-RT for participants in the highest quartile of mean heart dose (1787 mmHg% to 1732 mmHg%) but was stable or increased for participants in the first (1750 mmHg% to 1904 mmHg%), second (1937 mmHg% to 2114 mmHg%), and third (1883 mmHg% to 1880 mmHg%) quartiles. There was no correlation between mean or max heart dose with other myocardial work indices including GCW, GWW, or GWI.

### Reproducibility

Inter- and intra-observer variability for myocardial work indices as measured by ICC and standard error of measurement (SEM) are summarized in Table 4. GWI showed the best repeatability and reproducibility with an intra-observer and inter-observer ICC of 0.94 (95% CI: 0.87–0.97) and 0.94 (955 CI: 0.85–0.97), respectively, and an intra-observer and inter-observer SEM of 45.6 mmHg% and 47.4 mmHg%, respectively.

## Discussion

In this prospective study of women with breast cancer receiving multimodal treatment including regional nodal irradiation with VMAT, we observed no early reductions in echocardiographic parameters of LV systolic function including LVEF, GLS, or MW indices. The absence of significant changes in echocardiography-based measures of cardiac contractile function early after RNI with VMAT support further investigation of this contemporary RT technique which is being increasingly adopted. In addition, we found no correlation between mean or max cardiac RT dose and longitudinal changes in LVEF or GLS. Finally, we observed an association between mean heart dose and change in global work index from pre-RT to 6months post-RT. The utility of assessing myocardial work (in particular, GWI) to detect subclinical cardiotoxicity from breast RT may therefore warrant further investigation.

The cardiac implications of adjuvant treatment have been studied intently since the early days of postmastectomy RT. Indeed, owing to the limited precision of RT in prior eras, several reports raised the specter of significant RT-associated CV toxicity^2,3,7^. More recently, as the multidisciplinary management of breast cancer has improved in lockstep with radiotherapeutic technologies, several studies now support the increasing benefit of comprehensive RT for a variety of breast cancer presentations^11,12,24^. Two landmark trials, MA.20 and EORTC 22922, both demonstrated that RNI confers a significant 10-year disease-free survival benefit for those presenting even with a limited axillary disease burden. In parallel, the DBCG-IMN study affirmed the importance of including the internal mammary nodal (IMN) basin as a component of RNI, demonstrating an overall survival benefit when this region was encompassed in the radiotherapeutic target despite the elevated cardiac dose with this approach. Thus, an increasing proportion of breast cancer patients now receive RNI, along with the concomitant elevation in cardiac RT exposure, although without impinging on cardiac outcomes. And while none of these studies demonstrated elevated cardiac mortality with IMN coverage, the balance of literature to date suggests that increasing cardiac dose can only be detrimental to long term outcomes. To that end, the VMAT approach studied here is being increasingly employed for RNI, although as discussed above, lowering of the maximum heart dose as afforded by VMAT often comes at the expense of raising the mean heart dose with unclear implications that require further study.

Overall, the present findings are consistent with those from our previous study which showed no evidence of early subclinical LV systolic dysfunction after contemporary breast RT in women with HER2 positive breast cancer.^25^ We further extend these results in the current longitudinal cohort study with inclusion of patients treated with RNI using VMAT. Studies assessing the subclinical cardiotoxicity of RT have previously demonstrated radiation-induced global and segmental changes in myocardial strain and strain rate in patients with breast cancer, however breast radiotherapy in these studies was delivered using tangential photon beams with typical 3D-conformal dosimetry.^26–28^ More advanced breast radiotherapy techniques, such as VMAT, intensity modulated radiotherapy (IMRT), and proton therapy are now available and are increasingly used in standard care.

Myocardial work indices, which are measures of left ventricular mechanics that incorporate myocardial deformation with afterload and are less susceptible to fluctuations in blood pressure, have diagnostic and prognostic significance in several cardiovascular conditions including chemotherapy related cardiac dysfunction.^29,30^ This study further contributes to the current literature by assessing for changes in myocardial work in the setting of breast radiotherapy. Overall, no significant change in MW indices was observed in our patients throughout the radiotherapy treatment period. However, among participants in the highest quartile of mean heart dose, there was a decline in GWI. These findings possibly suggest that GWI can detect early subclinical changes in myocardial function related to radiotherapy. Further studies are needed to replicate these findings and determine whether GWI provides incremental diagnostic and prognostic value to established echocardiographic parameters.

These findings must be interpreted in the context of the study design. While our prospective cohort study was of limited size, it nonetheless provides novel data on the potential role of myocardial work for assessment of radiation-induced cardiotoxicity. Whether changes in myocardial work indices are associated with clinical cardiovascular outcomes will require larger studies with longer follow-up. In addition, most patients in this study were treated with anthracyclines and/or HER2-targeted therapies which can cause short- and long-term effects on cardiac function. Therefore, we cannot exclude the potential confounding effects of prior/concurrent breast cancer treatments on echocardiographic changes during radiotherapy. However, the absence of significant changes in LVEF or GLS within 6 months of radiation in the context of other cardiotoxic cancer treatment exposures suggest that radiation with VMAT does not enhance the cardiotoxic effects of other cardiotoxic breast cancer treatments.

In conclusion, in this small prospective study, VMAT for patients requiring regional nodal irradiation yielded no observable early decrement in echocardiographic parameters of cardiac function including LVEF and GLS. The cardiac implications of VMAT for patients requiring RNI merit further study, including among those receiving anthracyclines and HER2-directed therapy. Assessment of myocardial work indices such as GWI may be useful to identify subclinical cardiotoxicity in patients receiving among the highest allowable mean heart doses and warrants further consideration. Larger cohorts with longer follow-up will be needed to validate these findings.

## Funding/Support:

This study was supported by a grant from the Imaging and Radiation Sciences Program at Memorial Sloan Kettering Cancer Center, the Lois Green Fund, and in part by NIH/NCI Cancer Center Support Grant No. P30CA008748.

## Figures and Tables

**Table 1.** Patient and treatment characteristics

N = 19	median (age)
Age	38 (26–61)
Surgery	
Breast Conservation	3
Mastectomy	16
Sentinel node biopsy	4
Axillary lymph node dissection	15
Post-mastectomy reconstruction	12
Axillary nodes removed	16 (2–27)
Lymph nodes positive	2 (0–18)
Systemic therapy	
Neoadjuvant chemotherapy	15
Adjuvant chemotherapy	2
Doxorubicin	17
HER2-directed therapy	7
Trastuzumab	7
Pertuzumab	7
Endocrine therapy	13
Dosimetry	
Cardiac mean (cGy)	475 (187–698)
Cardiac max (cGy)	2999 (1560–4793)

**Table 2.** Longitudinal changes in conventional echocardiographic parameters during breast radiotherapy

	Pre-RT (n=19)	Post-RT (n=19)	6-month F/U (n=18)	P-value^1^
LVEDD/BSA	2.6 ± 0.3	2.6 ± 0.3	2.7 ± 0.2	0.818
LV mass index	74.7 ± 13.1	67.0 ± 15.4	68.3 ± 12.3	0.213
LA Volume index	25.3 ± 5.8	21.4 ± 6.6	22.2 ± 7.3	0.200
LVEF (%)				0.493
Median (IQR)	61 (58.5, 65.5)	62 (59.5, 65)	63 (61, 65)	
Range	54–69	55–72	53–68	
Mean ± SD	61.8 ± 4.4	62.2 ± 4.3	62.7 ± 3.8^†^	
Global longitudinal strain (%)	−19.3 ± 2.2	−19.2 ± 1.8	−19.6 ± 1.8	0.627
A3C	−18.9 ± 2.4	−19.6 ± 1.8	−19.7 ± 2.6	
A4C	−19.3 ± 2.5	−19.2 ± 2.1	−19.4 ± 2.0	
A2C	−19.7 ± 2.3	−19.2 ± 2.4	−19.8 ± 2.0	
Global circumferential strain (%)	−16.6 ± 3.6	−17.0 ± 2.7	−16.2 ± 3.5	0.986
Global radial strain (%)	45.8 ± 23.9	44.9 ± 10.4	43.3 ± 15.7	0.958
Diastolic Parameters				
Mitral E velocity (cm/sec)	77.3 ± 17.5	74.8 ± 15.7	77.2 ± 20.4	0.775
Mitral A velocity (cm/sec)	60.5 ± 16.3	61.7 ± 11.8	62.9 ± 13.8	0.648
Mitral E/A ratio	1.3 ± 0.4	1.3 ± 0.4	1.3 ± 0.4	0.417
Septal e’ (cm/sec)	10.3 ± 2.55	9.7 ± 3.0	10.0 ± 2.9	0.669
Lateral e’ (cm/sec)	13.0 ± 3.2	11.7 ± 2.9	12.1 ± 3.6	0.323
Septal E/e’ ratio	7.6 ± 1.9	8.0 ± 2.3	8.0 ± 1.5	0.274
Lateral E/e’ ratio	6.2 ± 1.7	6.5 ± 1.5	6.6 ± 1.2	0.191

Values are mean ± SD

1 Wilcoxon singed rank test between pre-RT and 6-month F/U (N=17)

**Table 3.** Longitudinal changes in myocardial work indices during breast radiotherapy

	Pre-RT (n=19)	Post-RT (n=19)	6-month F/U (n=18)	P-value^1^
Global work index, mmHg%	1834±238	1847 ± 238	1885±271	0.356
Global constructive work, mmHg%	1992±260	2026 ± 243	2073±226	0.093
Global wasted work, mmHg%	76 ± 39	71 ± 50	71 ± 28	0.287
Global work efficiency, %	96 ± 2	96 ± 2	96 ± 1	0.280

Values are mean ± SD

1 Wilcoxon singed rank test between pre-RT and 6-month F/U (N=17)

**Table 4.** Inter- and intra-observer variability for myocardial work indices

	Inter-observer variability			Intra-observer variability		
	ICC	95% CI	SEM	ICC	95% CI	SEM
Global work index (mmHg%)	0.94	0.867–0.974	45.64	0.936	0.853–0.972	47.43
Global constructive work (mmHg%)	0.899	0.79–0.953	55.35	0.909	0.795–0.961	56.08
Global wasted work (mmHg%)	0.849	0.661–0.937	14.5	0.829	0.64–0.923	14.2
Global work efficiency (%)	0.887	0.741–0.953	0.53	0.863	0.695–0.942	0.55

## Data Availability

The datasets used and/or analysed during the current study are available from the corresponding author on reasonable request.
